# Facilitating Effects of Reductive Soil Disinfestation on Soil Health and Physiological Properties of *Panax ginseng*

**DOI:** 10.1007/s00248-024-02349-4

**Published:** 2024-03-21

**Authors:** Yu Zhan, Ergang Wang, Yi Zhou, Guixiang He, Pengyuan Lv, Lixiang Wang, Tingting Zhou, Xinyue Miao, Changbao Chen, Qiong Li

**Affiliations:** 1 Jilin Ginseng Academy, Changchun University of Chinese Medicine, Changchun, 130117 China; 2 School of Pharmaceutical Sciences, Changchun University of Chinese Medicine, Changchun, 130117 China

**Keywords:** Soil disinfestation, Soil health, Fungal community, Physiological properties, *Panax ginseng*

## Abstract

Chemical soil fumigation (CSF) and reductive soil disinfestation (RSD) have been proven to be effective agricultural strategies to improve soil quality, restructure microbial communities, and promote plant growth in soil degradation remediation. However, it is still unclear how RSD and CSF ensure soil and plant health by altering fungal communities. Field experiments were conducted to investigate the effects of CSF with chloropicrin, and RSD with animal feces on soil properties, fungal communities and functional composition, and plant physiological characteristics were evaluated. Results showed that RSD and CSF treatment improved soil properties, restructured fungal community composition and structure, enhanced fungal interactions and functions, and facilitated plant growth. There was a significant increase in OM, AN, and AP contents in the soil with both CSF and RSD treatments compared to CK. Meanwhile, compared with CK and CSF, RSD treatment significantly increased biocontrol *Chaetomium* relative abundance while reducing pathogenic *Neonectria* relative abundance, indicating that RSD has strong inhibition potential. Furthermore, the microbial network of RSD treatment was more complex and interconnected, and the functions of plant pathogens, and animal pathogen were decreased. Importantly, RSD treatment significantly increased plant SOD, CAT, POD activity, SP, Ca, Zn content, and decreased MDA, ABA, Mg, K, and Fe content. In summary, RSD treatment is more effective than CSF treatment, by stimulating the proliferation of probiotic communities to further enhance soil health and plant disease resistance.

**Supplementary Information:**

The online version contains supplementary material available at 10.1007/s00248-024-02349-4.

## Introduction

Ginseng (*Panax ginseng* C. A. Meyer) is a popular medicinal herb, with rich nutritional value and medicinal effect [27]. It has been intensively cultivated in China and South Korea in recent years to meet the growing demand for production [66]. The availability of arable land for ginseng cultivation is limited, so farmers usually grow ginseng in the same soil continuously. Unfortunately, continuous monocropping of ginseng often accelerates soil quality degradation and soil-borne disease development, thereby posing a serious threat to plant health and damaging agriculture’s sustainable development [14, 18, 26]. Thus, an effective management practice is urgently needed to maintain crop productivity under highly intensive farming and continuous monocultures.

Chemical soil fumigation (CSF) is a common agricultural measure in current production, which has excellent control effects on soil-borne pathogens, pests, and weeds, but it is not selective and have significant toxic side effects on non-target microorganisms [9, 15, 16]. Therefore, the application of a large amount of chemical fumigates will cause external disturbances in the diversity and composition of microflora, which may affect the potential function of the soil ecosystem and cause ecological risks [33]. In addition, increasing concerns about food safety and environmental pollution have further limited chemical fumigation use [4]. Reductive soil disinfection (RSD), as an alternative approach to chemical soil disinfection, is generally considered a promising soil health management practice. By applying a large amount of easily decomposed organic materials to the soil and preventing air diffusion into the soil by means of irrigation and mulching, a strong anaerobic and reducing environment can be created in a short time to kill soil-borne pathogenic microorganisms, and improve soil quality [2, 64]. Until now, RSD has been widely applied in the field to successfully improving the soil quality of many crops, such as watermelon [38], cucumber [19], tomato [37]. However, these applications mainly focus on annual plants such as vegetables and fruits, while less research has been done on perennials.

Soil microorganisms are widely recognized as the main driving factors for soil functional stability and integrity [6, 28]. Fungi are critical components of the soil microbial system and play a vital role in soil structure formation, organic matter decomposition, and microecological balance [11, 61]. Plant rhizosphere is one of the most active hot spots of soil fungal community, and most root diseases are caused by fungi, such as *Cylindrocarpon destructans*, *Fusarium oxysporum*, *Botrytis cinerea Pers* [44, 53]. It has been shown that both CSF and RSD treatments significantly inhibited *Fusarium oxysporum* population and the genus *Fusarium* relative abundance [47, 70]. Therefore, changes in the diversity and composition of the fungal community are essential mechanisms in suppressing pathogens during soil disinfestation and are related to the health of the whole soil ecosystem [5, 10]. In addition, soil microbiomes are also crucial for plant growth, health, and diseases, promoting root nutrient absorption [3, 8], enhancing plant resistance to diseases [31], and improving salt resistance [7] and heavy metals [45]. Despite such promising results, how CSF and RSD affect other physiological properties of plants, particularly predictors related to plant defense responses, has not been well studied.

Plants create directional changes in soil microbiomes, which vary depending on the materials and functional groups used between CSF and RSD. Here, we hypothesized that (1) CSF and RSD differentially impact soil fungal community composition, structure and diversity; (2) CSF and RSD by changing soil fungal communities are conducive to managing plant physiological properties; and (3) RSD can reduce the number of pathogenic bacteria and promote plant growth, compared with CSF. To test these hypotheses, field experiment was conducted in a ginseng monoculture cropping system to investigate the effects of CSF and RSD on soil fungal diversity, soil nutrient content, and physiological properties of replanted seedlings.

## Materials and Methods

### Field Description and Experimental Design

Field experiments were conducted on August 20, 2019, in Zuojia Town, Changyi District, Jilin City, Jilin Province, China (44°02′N, 126°15′E, 237 m alt.). The region has a temperate continental monsoon climate, with an average annual temperature of 5.8 °C and precipitation of 550 mm, respectively. Ginseng had been consecutively cultivated for 3 years and suffered severe disease at this experimental site. The soil physicochemical properties of the experiment have been described previously [65].

Three treatments, (1) CK, untreated soil; (2) CSF, soil with 0.5 t∙ha^−1^ chloropicrin; and (3) RSD, soil with 15 t∙ha^−1^ animal feces, were performed with three replicates and each measured 30 m^2^ in a randomized complete block design. The chloropicrin was purchased from Dalian Lvfeng Chemical Co., Ltd. (Liaoning, China). The animal feces (chicken feces, cow feces, and pig feces = 1:1:1) were obtained from Zuojia Town, Changyi District, Jilin City, Jilin Province resident breeding farm (Jilin, China). The soil in CSF treatment was firstly irrigated 10 cm of chloropicrin, and then covered with 0.04-mm blue plastic film. The soil in RSD treatment was firstly added 10 cm of animal feces, irrigated to 100% water holding capacity, and then covered with 0.04-mm blue plastic film. All treatments except the control group were conducted under anaerobic conditions lasted for 4 weeks and the soil temperature was maintained at 30–40 °C. The plastic films were removed after 4 weeks and the soil was over-turned after 2–3 days of natural drying. Two-year-old healthy ginseng seedlings of similar size were transplanted on October 20, 2019.

### Sample Collection and Processing

Soil samples and plants from three treatments were collected during the harvesting period (October 1, 2020), and four replicates of each treatment were mixed as composite samples. The sampling depth was 0–20 cm. The collected soil samples were sieved and divided into two subsamples, one subsample was stored at 4 °C for physiochemical analyses, and another subsample was stored at −80 °C for the DNA analysis. Meanwhile, separate the aboveground and underground parts of the plant, wash gently, then dry the roots with absorbent paper and divide into two subsamples. One subsample was stored at −80 °C for physiological and biochemical analysis, and another subsample was dried at 45 °C for plant nutrient element analysis.

### Soil Physicochemical and Plant Physiological Properties

Soil organic matter (OM) was determined by the potassium dichromate external heating method [13]. Available nitrogen (AN) was determined using the alkali-hydrolyzed diffusion method [71]. Available phosphorus (AP) was extracted with NaHCO_3_ solution, and then molybdenum-antimony colorimetry [71].

Soluble protein content (SP) content was measured with Coomassie bright blue method with the measurement of absorbance at 595 nm. Malondialdehyde content (MDA) content was determined at 532 nm using 3,5,5′-trimethyloxazol-2,4-dione produced by the thiobarbituric acid reaction. The activities of plant superoxide dismutase (SOD), peroxidase (POD) and catalase (CAT) were measured using a kit produced by the company Nanjing Jiancheng Bioengineering Institute (Nanjing, China), and the activities were expressed in units per milligram of protein. Plant auxin (IAA), abscisic acid (ABA), and gibberellin (GA) contents were measured by enzyme-linked immunosorbent assay (ELISA). Plant nutrient elements Mg, K, Ca, Fe, Cu, and Zn were extracted by HNO_3_-HClO_4_ deboiling method and determined by ICP-OES (iCAP 7400 DUO, Thermofisher, USA). All measurements were conducted in quadruplicate to avoid random data.

### DNA Extraction and Miseq Sequencing

Total DNA was extracted from 0.5 g soil samples using an E.Z.N.A.® Soil DNA Kit. We used a NanoDrop 2000 spectrophotometer after extracting DNA to determine its quality and concentration. Agarose gel electrophoresis was used to validate DNA’s integrity. The hypervariable region of the fungi ITS gene was amplified with primer pairs ITS1F and ITS2R by an ABI GeneAmp® 9700 PCR thermocycler. Used the protocol described by Tan et al. [54] for the amplification of fungal ITS genes and analysis of PCR product purity. The diversity and composition of the microbial community were measured using the Illumina Miseq PE300 platform (Illumina USA) after purification. High-throughput sequencing results have been uploaded to NCBI (SRA Accession Number: PRJNA822700). FLASH (Version 1.2.11) was used to merge raw sequences generated by MiSeq paired-end sequencing. Used the UPARSE (Version 11) to cluster quality-filtered fungal sequences into operational taxa (OTUs) with 97% sequence similarity. Representative sequences were taxonomically classified using the Ribosomal Database Project (RDP) and then according to the Unite database, with a confidence threshold of 70% for fungi.

### Statistical and Bioinformatics Analysis

Using IBM SPSS 21.0 statistical software, we measured differences in soil physicochemical properties, and ginseng physiological characteristics between different treatments using one-way analysis of variance (ANOVA) (*P* < 0.05). Fungal α and β diversity was described by the Chao, Ace, Shannon, and Simpson indices, and estimated with QIIME 2 software. The Bray-Curtis distance was used for PCoA and hierarchical cluster analysis. Linear discriminant analysis (LDA) effect size (LEfSe) was used to identify taxonomic fungal taxa among different treatments. Microbial networks were constructed using R (Version 4.2.3) software and visualized using Gephi (Version 0.92). Correlation coefficients |*r*| < 0.6 and *P* > 0.05 of the correlation R matrix were removed. The FUNGuild databases were used to predict fungal communities’ functional compositions. Heat map correlation analysis was used to visualize the relationships between dominant genera, soil physicochemical properties, and plant physiological properties.

## Results

### Soil Physicochemical Properties

Analysis of the collected soil samples showed a clear difference in physicochemical properties changes after RSD and CSF treatment of ginseng planted soil (*P* < 0.05, Table 1). Both RSD and CSF treatment increased soil OM, AN, and AP content compared with untreated soil (CK). In particular, the content of OM and AN was highest in the RSD treatment, and the content of AP was highest in the CSF treatment.

**Table 1:** Soil physicochemical under different treatments

Treatment	OM (g∙kg^−1^)	AN (mg∙kg^−1^)	AP (mg∙kg^−1^)
CK	58.04 ± 5.06 c	160.30 ± 2.06 c	19.23 ± 0.10 c
CSF	70.65 ± 2.14 b	243.13 ± 4.05 b	22.33 ± 0.37 a
RSD	79.94 ± 2.81 a	267.63 ± 8.01 a	20.51 ± 0.05 b

The one-way ANOVA indicates significant differences at *P* < 0.05 between values (mean SD, *n* = 4) within the same column followed by different letters

### Soil Microbial α and β Diversities

A total of 843,706 high-quality ITS sequences were obtained from 12 soil samples (3 treatments × 4 biological replicates), with sequences ranging from 67,955 to 73,521 per sample. After re-sampling, the minimum number of sample sequences is used for leveling, and then classified into 1096 OTUs at 97% sequence identity. The sequence depth in the present study was sufficient for diverse analyses as revealed that the coverage of all samples was above 99.7% (Supplemental Table S1).

**Fig. 1:**
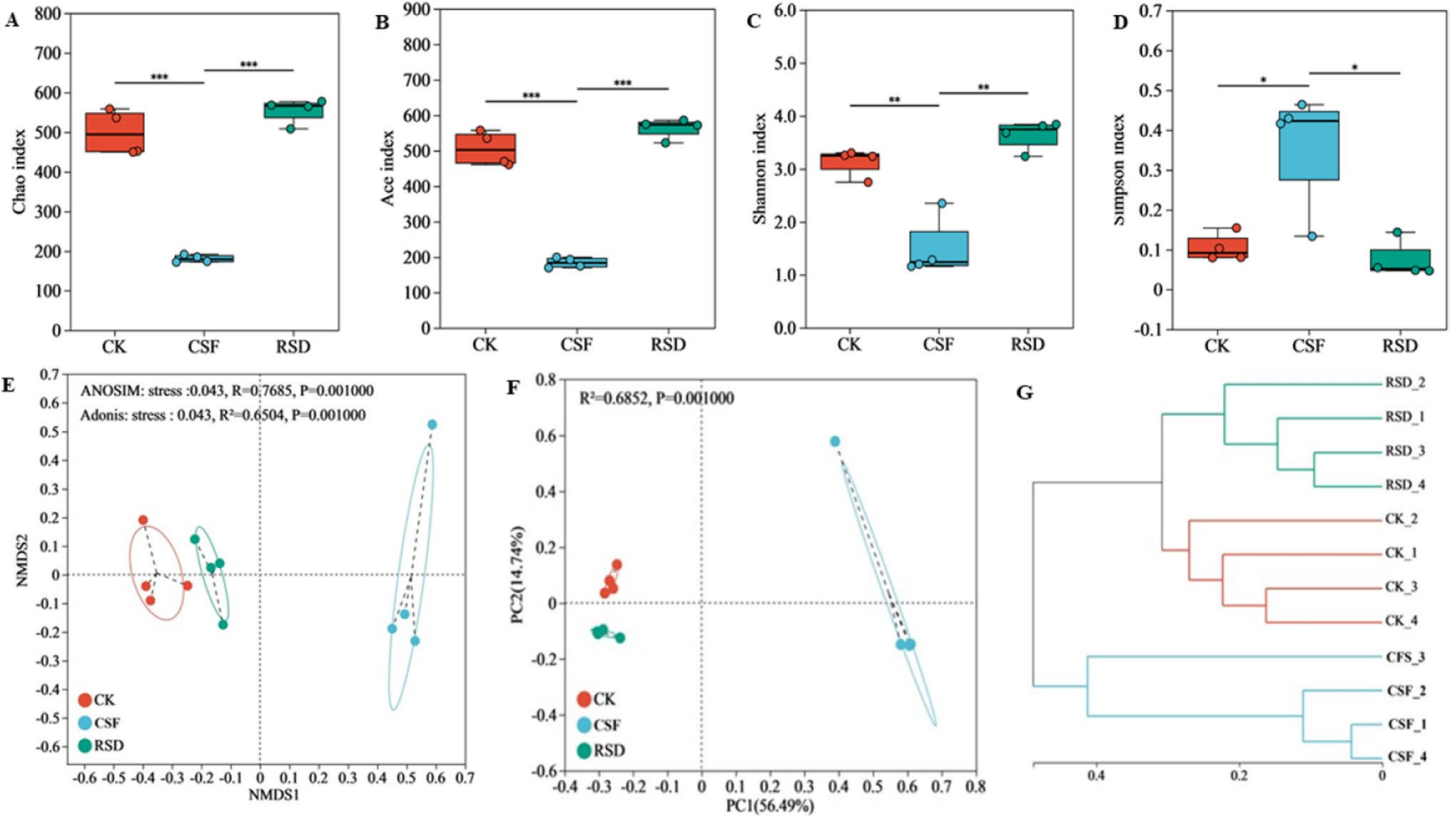
Soil fungal richness and diversity under different soil disinfection treatments (**A**–**D**). Non-metric multidimensional scaling analysis (**E**), principal coordinate analysis (**F**) and hierarchical cluster analysis (**G**) based on Bray-Curtis distance were conducted for fungal communities OTU in different soil samples. The Chao index and Ace index represent fungal diversity, and Shannon index and Simpson index represent species richness. The symbols *, **, and *** indicate significant differences at *P* < 0.05, *P* < 0.01, and *P* < 0.001 according to the one-way ANOVA, respectively

We found that species richness and diversity of fungal communities in CSF-treated soil versus RSD-treated soil differed significantly (Fig. 1A–D). The species richness (Chao index and Ace index) and fungal diversity (Shannon index and Simpson index) of RSD treatment were significantly higher than CSF treatment, but there was no significant difference between RSD treatment and CK treatment. In contrast, the species richness and fungal diversity of CSF-treated soils significantly decreased compared to CK treatment.

NMDS plots visualize fungal β-diversity patterns at genus level with a stress value of 0.043 (Fig. 1E–G). The pattern of rhizosphere fungal community is clearly distinguished between the horizontal axis and the vertical axis, in which the microbial community of RSD treatment is separated from that of CSF treatment. The fungal community assemblage at genus level between different treatments explained the evident difference in treatment effect calculated by PERMANOVA test (ADONIS R^2^ 0.6504 and ANOSIM R^2^ 0.7685; *P* < 0.001) (Supplemental Table S2,3). Similarly, PCoA showed that the dissimilarity between rhizosphere fungal communities at species level (56.49% explained by PCo1) was largely due to RSD and CSF microbial samples. In addition, Bray-Curtis clustering analysis separated the distribution of microbial communities (Fig. 1G), where the fungal communities of RSD treated microbial samples were clustered together and separated from those of CSF treated samples.

### Soil Microbial Communities Compositions and Potential Function Prediction

**Fig. 2:**
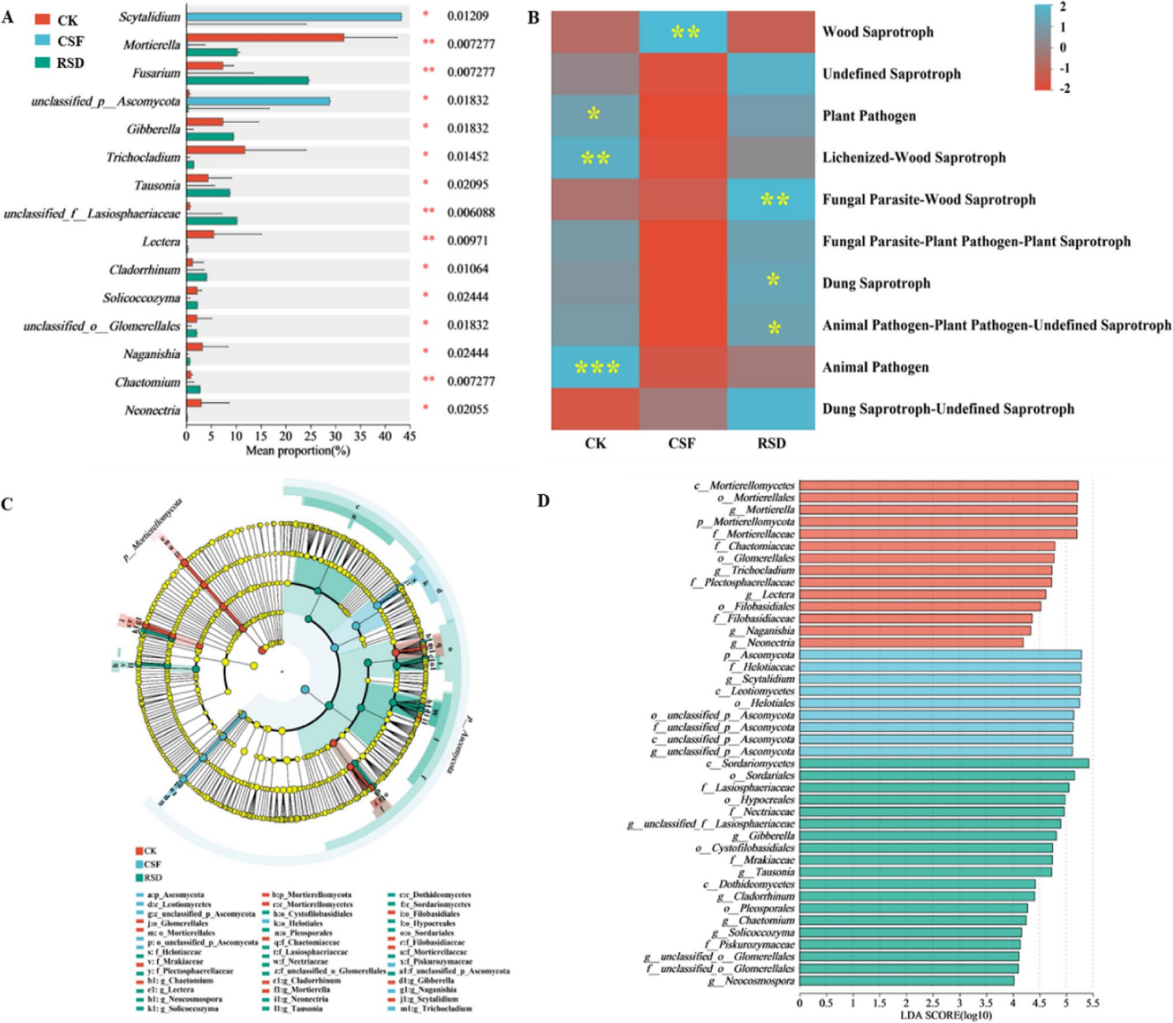
Relative abundance of top 15 fungal genera (**A**) and functional groups in fungi (**B**) in soil samples from different treatments; linear discriminant analysis shows the differences of fungal taxa (**C**) (from phylum to genus) among different treatments, and the significantly enriched fungal taxa (**D**) among different treatments. The symbols *, **, and *** behind the taxa indicate significant differences at *P* < 0.05, *P* < 0.01, and *P* < 0.001; red and blue colors indicate the negative and positive correlations, respectively. Taxa with significant differences in abundance (LDA score > 4, *P* < 0.05) between different treatments are colored

At phylum level (Fig. S1A-B), the rhizosphere community structure of fungi is classified into the top 3 phyla, accounting for over 98% of the total fungal sequence. Among them, *Ascomycota* was the most abundant, accounting for 51.78–92.49% that mostly enriched in CSF treatments. Similarly, *Mortierellomycota* relative abundance decreased after CSF, and RSD applications, respectively. As compared to CK treatment, fungal genera indicated that the proportion of biocontrol *Chaetomium* taxa significantly increased and pathogenic *Neonectria* significantly decreased during RSD treatment (Fig. 2A). The proportion of biocontrol *Chaetomium* significantly increased by 99.6, under RSD soil compared to CSF. In addition, LEfSe analysis revealed that the CSF and RSD treatments significantly altered the fungal communities from the phyla to the genus and that different treatments harbored distinct biomarkers (Fig. 2C, D). For instance, the taxa *Ascomycota*, *Helotiaceae*, *Scytalidium*, *Leotiomycetes*, and *Helotiales* were significantly enriched in CSF soil, *Sordariomycete*s, *Sordariale*s, *Gibberella*, *Lasiosphaeriaceae*, *Hypocreales*, *Nectriaceae*, *Cystofilobasidiales*, *Mrakiaceae*, *Tausonia*, *Dothideomycetes*, *Cladorrhinum*, *Pleosporales*, *Chaetomium*, *Solicoccozyma*, *Piskurozymaceae*, and *Neocosmospora* were significantly enriched in RSD soil.

Overall, a total of three fungal trophic modes (pathotroph, symbiotroph, and saprotroph) were found. RSD-treated soil exhibited significantly enriched relative abundances of fungi functional groups associated with dung saprotrophs, fungal parasites-wood saprotrophs, and dung saprotroph, fungal parasite-wood saprotroph, fungal parasite-wood saprotroph, and dung saprotroph-soil saprotroph, compared to CK-treated soil (Fig. 2B). Notably, the relative abundance of fungal plant pathogens, lichenized-wood saprotroph, and animal pathogen was decreased in RSD-treated soil (Fig. 2B).

### Co-occurrence Networks of Microbial Communities

**Fig. 3:**
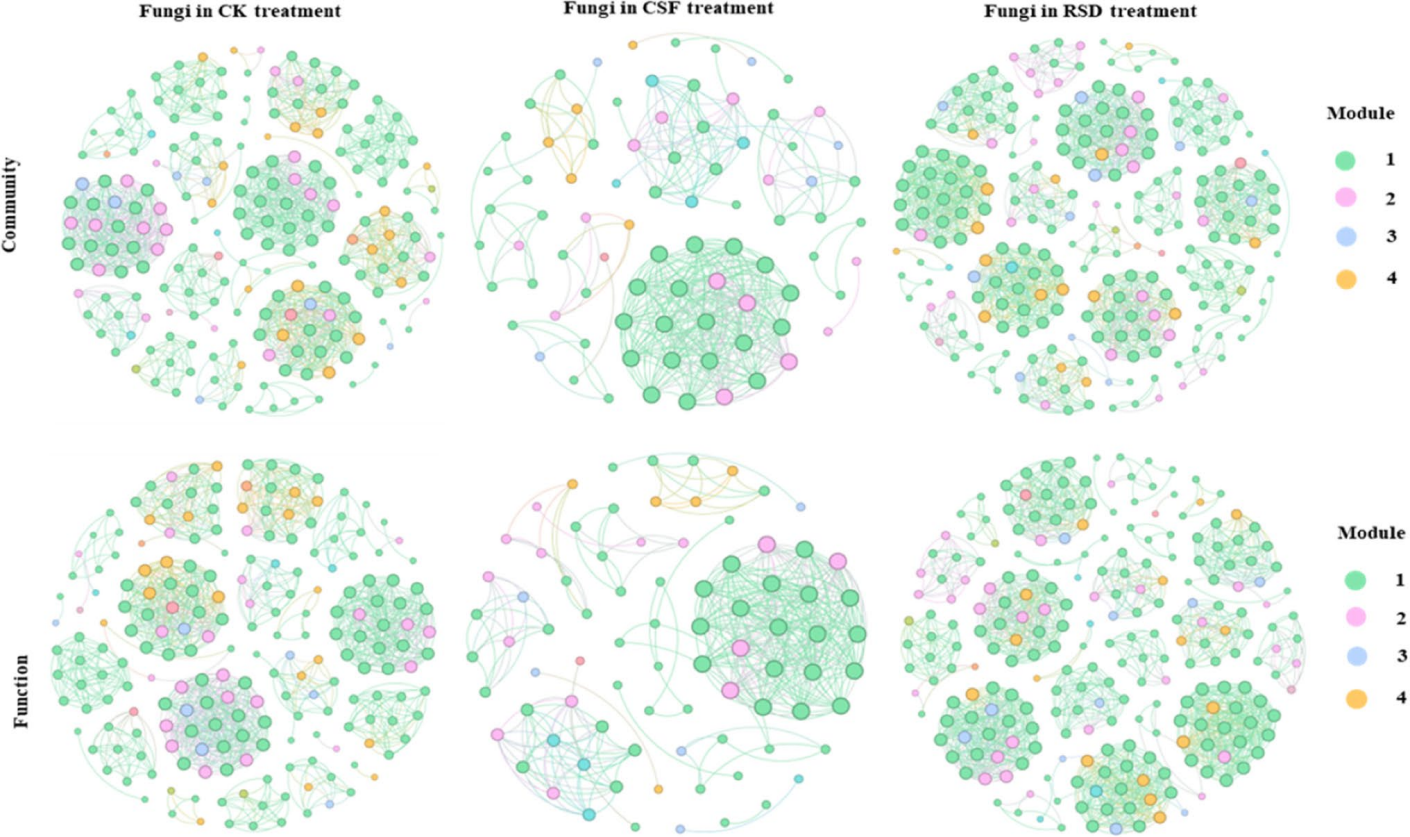
Co-occurrence networks of fungal communities and functions under different treatments. The keystone taxa were marked in each network

We found clear differences in fungal community networks, and topological characteristics, between different treatments (Fig. 3, Table 2). RSD-treated soil, for example, had more nodes and edges, more modularity, longer average paths, and more weighted degrees of fungal network (Table 2). All the nodes with Zi ≥ 2.5 or Pi ≥ 0.62 were determined as the keystone species, that is, nodes in the area of connectors (0.24%), module hubs (0.65%), and network hubs (0%) played a crucial role in the co-occurrence networks (Fig. S2).

**Table 2:** Topological characteristics of fungal community networks in different treatments

Topological characteristics	CK	CSF	RSD
Number of nodes	203	82	235
Number of edges	1205	362	1436
Modularity	0.873	0.563	0.895
Average path length	1.165	1.203	1.233
Average weighted degree	11.872	8.829	12.221

In addition, there were significant differences in the identity of the top ten fungal keystone taxa between different treatments. For example, the fungal keystone taxa *Trichocladiu*, *Lectera*, and *Neonectria* were found in CK treatment, *Scytalidium*, *Byssochlamys*, *Cutaneotrichosporon*, *Issatchenkia*, *Thermomyces*, *Thermoascus*, and *Monascus* were found in CSF treatment, whereas *Gibberella*, *Cladorrhinum*, and *Solicoccozyma* were found in RSD treatment (Table S4).

### Plant Physiological Properties

Analysis of the collected ginseng samples showed a clear difference in the physiological properties of ginseng treated with RSD and CSF (*P* < 0.05, Table 3). Compared with untreated ginseng (CK), ginseng SOD, CAT, POD activity and SP, Ca, Zn content were increased, and ginseng MDA, ABA, Mg, K, and Fe content were decreased by RSD treatment. However, ginseng SOD, CAT activity and Mg, Ca content were decreased, and ginseng IAA content was increased by the CSF treatment. Furthermore, GA and Cu contents did not differ significantly between treatments.

**Table 3:** Plant physiological properties under different treatments

Treatment	CK	CSF	RSD
SP (μg∙mL^−1^)	84.762 ± 0.924 b	83.084 ± 0.466 b	89.274 ± 0.778 a
SOD (U∙mgprot^−1^)	39.538 ± 4.162 b	26.536 ± 1.874 c	51.124 ± 1.233 a
MDA (nmol∙mgprot^−1^)	0.454 ± 0.012 a	0.405 ± 0.006 b	0.423 ± 0.004 b
POD (U∙mgprot^−1^)	1.157 ± 0.025 b	1.053 ± 0.016 b	2.597 ± 0.079 a
CAT (U∙mgprot^−1^)	0.387 ± 0.005 b	0.314 ± 0.016 c	0.555 ± 0.037 a
GA (pmol∙mL^−1^)	122.465 ± 2.376 a	124.302 ± 3.747 a	118.040 ± 2.319 a
IAA (μmol∙L^−1^)	22.142 ± 0.374 b	28.133 ± 0.401 a	21.149 ± 0.901 b
ABA (ng∙mL^−1^)	131.064 ± 3.087 a	126.225 ± 0.482 a	103.133 ± 1.997 b
Mg (mg∙g^−1^)	1.046 ± 000 a	0.843 ± 0.082 b	0.968 ± 0.082 ab
Cu (mg∙g^−1^)	0.005 ± 0.001 a	0.003 ± 0.001 a	0.003 ± 0.000 a
Ca (mg∙g^−1^)	1.528 ± 0.082 ab	1.401 ± 0.082 b	1.649 ± 0.082 a
Fe (mg∙g^−1^)	0.163 ± 0.001 a	0.136 ± 0.000 b	0.123 ± 0.000 c
K (mg∙g^−1^)	7.648 ± 0.082 a	6.858 ± 0.082 b	5.158 ± 0.082 c
Zn (mg∙g^−1^)	0.036 ± 0.001 c	0.046 ± 0.001 b	0.121 ± 0.000 a

The one-way ANOVA indicates significant differences at *P* < 0.05 between values (mean SD, *n* = 4) within the same column followed by different letters

### Relationships Between Soil Physicochemical Properties, Plant Physiological Properties, and Microbial Communities

**Fig. 4:**
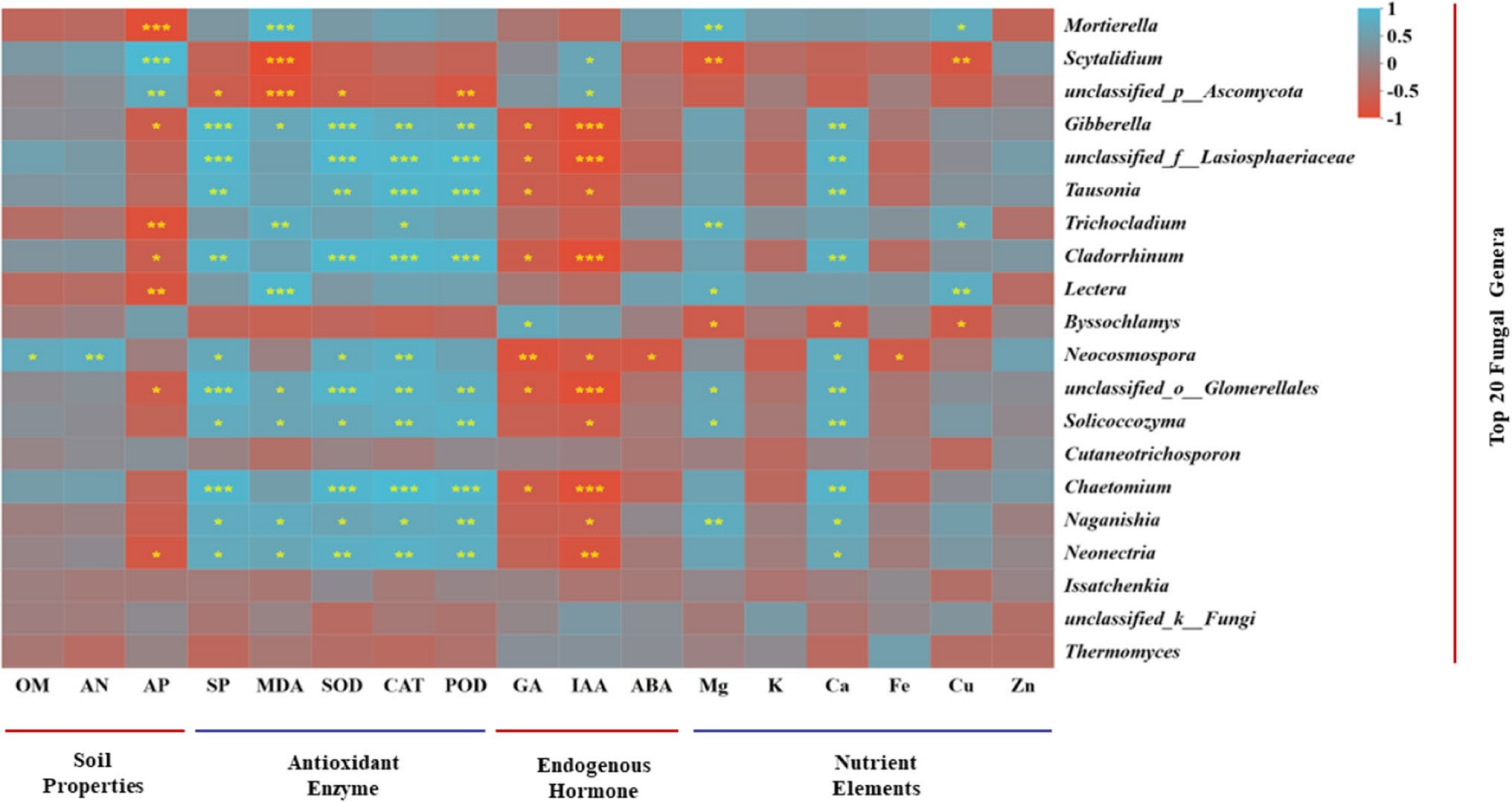
Correlations between dominant genera and soil physicochemical properties, plant physiological properties. The symbols *, **, and *** behind the taxa indicate significant differences at *P* < 0.05, *P* < 0.01, and *P* < 0.001; red and blue colors indicate negative and positive correlations, respectively

Most of the dominant genera which the relative abundance in CSF and RSD treated soil was significantly (*P* < 0.05) correlated with soil physicochemical properties, and plant physiological properties (Fig. 4). *Gibberella*, and *Cladorrhinum* relative abundances were significantly (*P* < 0.05) and positively correlated with SP, Ca content, SOD, CAT, and POD activity, and negatively correlated with AP, GA, and IAA content (Fig. 4). *Mortierella*, *Lectera*, and *Trichocladium* relative abundances were significantly (*P* < 0.05) and positively correlated with MDA, Mg, and Cu content, and negatively correlated with AP content (Fig. 4). *Scytalidium* relative abundance was significantly (*P* < 0.05) and positively correlated with AP, and IAA content, and negatively correlated with MDA, Mg, and Cu content (Fig. 4). *Tausonia* relative abundance was significantly (*P* < 0.05) and positively correlated with SP, Ca content, SOD, CAT, and POD activity, and negatively correlated with GA, and IAA content (Fig. 4). *Byssochlamys* relative abundance was significantly (*P* < 0.05) and positively correlated with GA content, and negatively correlated with Mg, Ca, and Cu content (Fig. 4). *Neocosmospora* relative abundance was significantly (*P* < 0.05) and positively correlated with OM, AN, SP, GA content, SOD, and CAT activity, and negatively correlated with GA, IAA, and ABA content (Fig. 4). *Chaetomium*, *Solicoccozyma*, *Naganishia*, and *Neonectria* relative abundances were significantly (*P* < 0.05) and positively correlated with SP, and Ca content, SOD, CAT, and POD activity, and negatively correlated with IAA content (Fig. 4).

## Discussion

### RSD Combined with Organic Materials Can Effectively Restore Soil Health and Functions

It is well known that soil is an extremely complex ecosystem, and unfortunately, soil has rapidly degraded globally as a result of continuous monocultures in intensive agriculture [32, 68]. To overcome this problem, solarization, crop rotation, intercropping, and antagonist introduction have generally been adopted in cultivation [50, 60]. However, these practices alone do not always eliminate the negative effects of continuous single cultivation, which are either restricted by environmental conditions or hampered by unstable results. Soil organic matter content is considered one of the important indicators for evaluating soil fertility and soil quality, which can promote plant growth and development, nutrient decomposition, and improve soil properties [49, 56]. In the present study, the OM content was considerably increased in both SFC and RSD treatments, with the greatest increase in OM found during RSD treatment, as reported in previous studies [20, 51]. Additionally, the effects of addition of decomposable carbon source and irrigation on the transformation of large amounts of nutrients are not consistent in different studies. As reported in the present study, the AN and AP content was considerably increased in both SFC and RSD treatments, with the highest content observed in the RSD treatment. The increase in macronutrient availability might result directly from the anaerobic degradation of animal feces or indirectly from the enhancement of nutrient cycling [48]. The abundance, diversity, and functional composition of microorganisms have long been considered important predictors of soil health [29, 46]. It is well known that soil fungi are an integral component of the soil microbial system, contributing to soil structure formation, fertility improvement, and microecological balance, participating in a wide variety of ecological processes [16, 43]. Many studies have found that RSD and SFC treatments can alter soil fungal communities and enhance their resistance to soil-borne pathogen invasion [34, 39, 70]. Similar to these studies, we found that both RSD and SFC treatments significantly reduced known soil-borne pathogens *Neonectria* and increased known disease-suppressive agents *Chaetomium* relative abundance, but the changes in RSD treatments were greater than those in SFC treatments. For example, *Chaetomium* can inhibit the growth of pathogenic fungi and promote soil nutrient activity by producing cellulase and chaetomin [1, 24].

There is no doubt that SFC and RSD treatment regulate the soil microbial community and suppress soil-borne pathogens, but their effects on other soil functions do not yet appear to be fully understood. We found that RSD significantly decreased microbial functions associated with fungal plant pathogens, lichenized-wood saprotroph, and animal pathogen. This is because the presence of antifungal compounds through the decomposition of different organic substrates during RSD treatment may inhibit fungal taxa growth [22, 40]. Likewise, according to previous studies, the fungi networks of healthy soils are more complex than those of diseased soils, indicating that fungi network characteristics can play a major role in predicting plant health [42, 59]. The present study revealed that both the complexity and connectivity of fungi networks were greater in RSD treatment soils, indicating that RSD treatment can effectively improve soil microbial ecosystem stability.

### RSD Combined with Organic Materials Further Enhanced the Performance of Plant Physiological Properties

Maintaining soil health is considered an important prerequisite for successfully alleviating replanting failures, as soil factors can affect the physiological and biochemical processes of the soil and affect the physiological characteristics of plants [36, 41]. As already discussed, RSD treatment can significantly increase soil AN and AP content. Alterations in soil nutrient availability may cause imbalances in plant nutrients [12]. This study found that RSD treatment significantly increased Ca and Zn content in ginseng roots and reduced Mg, K, Fe content, while the SFC treatment reduced Mg and Ca contents. These findings are encouraging since these nutrients form part of essential proteins and complexes in the plant, so their deficiency can compromise the physiological balance of the plant and the root activity [17].

Previous studies have found that when plants are subjected to stress, the reactive oxygen species and free radicals in their bodies become imbalanced, causing damage to the cell membrane system and inhibiting plant growth [67]. This study found that RSD treatment significantly increased ginseng roots’ SOD, CAT, and POD activities, while reducing MDA content. It may be because the decrease in MDA content after RSD treatment promotes the activity of protective enzymes such as SOD, CAT, and POD, converting toxic H_2_O_2_ into H_2_O, leading to a dynamic balance of SOD dominated disproportionation reactions and reducing cell membrane damage [35, 41, 52]. Moreover, soluble proteins, as an important osmotic regulator in plants, can also affect plant disease resistance by participating in various intracellular enzymatic reactions [30]. From the present study, we found interesting results that RSD-treated soils appeared to contain substantial increases in TP content, which may contribute to systemic resistance in plants.

As important signaling molecules, endogenous hormones play a crucial role in regulating plant growth and development, coping with biotic and abiotic stressors, maintaining homeostasis, and adapting to environmental changes in plants [25, 55, 57, 58]. The study demonstrated that RSD treatment significantly reduced the IAA and ABA content of ginseng roots, while there was no significant difference in GA content between different treatments. This is because ABA and IAA play an important role in stress response as a signal regulator in the stress chain, and RSD treatment can protect ginseng roots from stress in a way that does not require altering hormone levels to regulate stomatal closure in order to enhance stress resistance [55].

### Linking the Reassembled Soil Microbiomes with Soil Physicochemical Properties and Plant Physiological Properties

Previous studies suggested that EC, pH, OM, UE, SC, and ACP are the key factors affecting soil microbial communities [63, 69]. This study found significant relationships between soil OM, AN, and AP contents and microbial taxa. Soil OM and AN are considered a mobile, important C and N sources for microorganisms, 10–40% of them can easily be used by microorganisms between days and months [23]. This is consistent with the significant positive correlation between OM and AN content related to C and N decomposition and some microorganisms (i.e., *Firmicutes*). Meanwhile, *Mortierella* is a potential plant pathogen [21]. Many studies have shown that SFC and RSD practices could effectively reduce soil-borne pathogens [51, 62], which was in line with our study that *Mortierella* abundance was significantly reduced in all SFC and RSD treatments. Likewise, soil AP was also significantly negative correlated with *Mortierella* abundance, which also verified the above results.

As one of the most active hot zones for soil microorganisms, the rhizosphere of plants not only has extremely complex interactions with microorganisms, but also integrates the interactions between microorganisms and plants [58]. It is not surprising then, that RSD improves plant health performance primarily by improving microbial communities. Here, plant physiological differences were significantly related to dissimilarities in the relative abundance of the dominant fungal genera. For example, *Mortierella*, *Lectera*, and *Trichocladium* relative abundances were significantly and positively correlated with MDA, Mg, and Cu content. As a result of these results, it has been shown that the soil disinfestation and plant protection processes of RSD treatment are primarily mediated by fungal community. Additionally, physiological characteristics of ginseng roots were more significantly affected by RSD treatment than by SFC treatment. Such discrepancies could be attributed to differences in the chemistry (i.e., degradability) and quantity (i.e., availability) of C sources in the organic materials used [40, 54].

## Conclusion

Chemical soil fumigation and reductive soil disinfestation can considerably alleviate plant replant failure through reorganizing fungal communities and repairing the soil environment. However, the effect of reductive soil disinfestation is better than that of chemical soil fumigation. In particular, the soil microbiome was rebalanced by an increase in the abundance of beneficial taxa and a decrease in the abundance of pathogenic taxa. Meanwhile, the microbial network processed by RSD is more complex and interrelated, and reduces the function of plant pathogens. Furthermore, RSD treatment also changed soil properties, especially increased soil OM, AN, AP contents. Importantly, RSD treatment significantly increased plant SOD, CAT, POD activity, SP, Ca, Zn content, and decreased MDA, ABA, Mg, K, and Fe content. Thus, RSD practice may not only improve soil quality, change microbial community structure, inhibit pathogenic bacteria proliferation but also contribute to the growth of replanted crops, a potential agricultural practice.

### Supplementary Information

Supplementary file 1

## Data Availability

The raw sequencing data were deposited at the NCBI Sequence Read Archive database with the accession number of PRJNA822700.

## References

[1] Abdel-Azeem A, Gherbawy Y, Sabry AM (2016). Enzyme profiles and genotyping of *Chaetomium globosum* isolates from various substrates. Plant Biosyst.

[2] Ali A, Elrys AS, Liu LL, Xia Q, Wang BY, Li YL, Dan XQ, Iqbal M, Zhao J, Huang XQ, Cai ZC (2023). Deciphering the synergies of reductive soil disinfestation combined with biochar and antagonistic microbial inoculation in cucumber fusarium wilt suppression through rhizosphere microbiota structure. Microb Ecol.

[3] Begum N, Qin C, Ahanger MA, Raza S, Khan MI, Ashraf M, Ahmed N, Zhang LX (2019). Role of arbuscular mycorrhizal fungi in plant growth regulation: implications in abiotic stress tolerance. Front Plant Sci.

[4] Bell CH (2000). Fumigation in the 21st century. Crop Prot.

[5] Benli̇oğlu S, Yildiz A, Döken T (2004). Studies to determine the causal agents of soil-borne fungal diseases of strawberries in Aydin and to control them by soil disinfestation. J Phytorathol.

[6] Berendsen RL, Pieterse CMJ, Bakker PAHM (2012). The rhizosphere microbiome and plant health. Trends Plant Sci.

[7] Castrillo G, Teixeira PJPL, Paredes SH, Law TF, Lorenzo LD, Feltcher ME, Finkel OM, Breakfield NW, Mieczkowski P, Jones CD, Paz-Ares J, Dang JL (2017). Root microbiota drive direct integration of phosphate stress and immunity. Nature..

[8] Chen ZJ, Jin YY, Yao X, Chen TX, Wei XK, Li CJ, White JF, Nan ZB (2020). Fungal endophyte improves survival of *Lolium perenne* in low fertility soils by increasing root growth, metabolic activity and absorption of nutrients. Plant Soil.

[9] Dangi SR, Gerik JS, Tirado-Corbalá R, Ajwa H (2015). Soil microbial community structure and target organisms under different fumigation treatments. Appl Environ Soil Sci.

[10] de los Santos B, Medina JJ, Miranda L, Gómez JA, Talavera M (2021). Soil disinfestation efficacy against soil fungal pathogens in strawberry crops in Spain: an overview. Appl Agron J.

[11] Devi R, Kaur T, Kour D, Rana KL, Yadav A, Yadav AN (2020). Beneficial fungal communities from different habitats and their roles in plant growth promotion and soil health. Microbial Biosystems.

[12] Díaz-López M, Siles JA, Ros C, Bastida F, Nicolás (2022) The effects of ozone treatments on the agro-physiological parameters of tomato plants and the soil microbial community. Sci Total Environ 812:151429 10.1016/j.scitotenv.2021.151429 34742984

[13] Fan DQ, Zhang YQ, Qin SG, Wu B (2017). Relationships between *Artemisia ordosica* communities and environmental factors following sand-dune stabilization in the Mu Us desert, northwest China. J Forestry Res.

[14] Goodwin PH (2022). The rhizosphere microbiome of ginseng. Microorganisms.

[15] Guo HC, Zhao X, Rosskopf EN, Gioia FD, Hong JC, McNear Jr DH (2018) Impacts of anaerobic soil disinfestation and chemical fumigation on soil microbial communities in field tomato production system. Appl Soil Ecol 126:165–173

[16] Guo JJ, Ling N, Chen ZJ, Xue C, Li L, Liu LS, Gao LM, Wang M, Ruan JY, Guo SW, Vandenkoornhuyse P, Shen QR (2020) Soil fungal assemblage complexity is dependent on soil fertility and dominated by deterministic processes. New Phytol 226:232–243 10.1111/nph.16345 31778576

[17] Hänsch R, Mendel RR (2009) Physiological functions of mineral micronutrients (Cu, Zn, Mn, Fe, Ni, Mo, B, Cl). Curr Opin Plant Biol 12:259–266 10.1016/j.pbi.2009.05.006 19524482

[18] Huang LF, Song SX, Xia XJ, Mao WH, Shi K, Zhou YH, Yu JQ (2013). Plant-soil feedbacks and soil sickness: from mechanisms to application in agriculture. J Chem Ecol.

[19] Huang XQ, Liu LL, Wen T, Zhang JB, Wang FH, Cai ZC (2016). Changes in the soil microbial community after reductive soil disinfestation and cucumber seedling cultivation. Appl Microbiol Biotechnol.

[20] Huang XQ, Liu LL, Wen T, Zhu R, Zhang JB, Cai ZC (2015). Illumina MiSeq investigations on the changes of microbial community in the *Fusarium oxysporum f.sp. cubense* infected soil during and after reductive soil disinfestation. Microbiol Res.

[21] Huang XQ, Liu SZ, Liu X, Zhang SR, Li L, Zhao HT, Zhao J, Zhang JB, Cai ZC (2020). Plant pathological condition is associated with fungal community succession triggered by root exudates in the plant-soil system. Soil Boil Biochem.

[22] Huang XQ, Zhao J, Zhou X, Zhang JB, Cai ZC (2019). Differential responses of soil bacterial community and functional diversity to reductive soil disinfestation and chemical soil disinfestation. Geoderma..

[23] Ji CY, Ye RZ, Yin YF, Sun XF, Ma HL, Gao R (2022). Reductive soil disinfestation with biochar amendment modified microbial community composition in soils under plastic greenhouse vegetable production. Soil Tillage Res.

[24] Jiang C, Song J, Zhang J, Yang QB (2017). New production process of the antifungal chaetoglobosin A using cornstalks. Braz J Microbiol.

[25] Jin MR, Liu YL, Shi BS, Yuan H (2023) Exogenous IAA improves the seedling growth of Syringa villosa via regulating the endogenous hormones and enhancing the photosynthesis. Sci Hortic-Amsterdam 308:111585

[26] Jin Q, Zhang YY, Ma YY, Sun H, Guan YM, Liu ZB, Ye Q, Zhang Y, Shao C, Mu P, Wang QX (2022). The composition and function of the soil microbial community and its driving factors before and after cultivation of *Panax ginseng* in farmland of different ages. Ecol Indic.

[27] Karmazyn M, Gan XT (2021). Chemical components of ginseng, their biotransformation products and their potential as treatment of hypertension. Mol Cell Biochem.

[28] Kennedy AC, Smith KL (1995). Soil microbial diversity and the sustainability of agricultural soils. Plant Soil.

[29] Kennedy AC, Stubbs TL (2006). Soil microbial communities as indicators of soil health. Ann Arid Zone.

[30] Kruijt M, De Kock MJD, De Wit PJ (2005). Receptor-like proteins involved in plant disease resistance. Mol Plant Pathol.

[31] Kwak MJ, Kong HG, Choi K, Kwon SK, Guan YM, Song JY, Lee J, Lee PA, Choi SY, Seo M, Lee HJ, Jung EJ, Park H, Roy N, Kim H, Lee MM, Rubin EM, Lee SW, Kim JF (2018). Rhizosphere microbiome structure alters to enable wilt resistance in tomato. Nat Biotechnol.

[32] Li B, Zhou J, Lu Y, Xiong ZQ (2019). Field-aged biochar reduces the greenhouse gas balance in a degraded vegetable field treated by reductive soil disinfestation. Environ Sci Pollut R.

[33] Li J, Chen Y, Qin XY, Cao A, Lu A (2022). Impact of biochar on rhizosphere bacterial diversity restoration following chloropicrin fumigation of planted soil. Int J Environ Res Public Health.

[34] Li X, Li XF, Li YY, Dai XZ, Zhang QZ, Zhang M, Zhang ZQ, Tao Y, Chen WC, Zhang MM, Zhou XY, Yang S, Ma YQ, Zhran M, Zou XX (2021). Improved immobilization of soil cadmium by regulating soil characteristics and microbial community through reductive soil disinfestation. Sci Total Environ.

[35] Li Y, Han LX, Wang BB, Zhang J, Nie JY (2022). Dynamic degradation of penconazole and its effect on antioxidant enzyme activity and malondialdehyde content in apple fruit. Sci Hortic-Amsterdam.

[36] Li YL, Wang BY, Chang YF, Yang YT, Yao CZ, Huang XQ, Zhang JB, Cai ZC, Zhao J (2019). Reductive soil disinfestation effectively alleviates the replant failure of Sanqi ginseng through allelochemical degradation and pathogen suppression. Appl Microbiol Biotechnol.

[37] Liao H, Fan HX, Li YY, Yao HY (2021). Influence of reductive soil disinfestation or biochar amendment on bacterial communities and their utilization of plant-derived carbon in the rhizosphere of tomato. Appl Microbiol Biotechnol.

[38] Liu LL, Chen SH, Zhao J, Zhou X, Wang BY, Li YL, Zheng GQ, Zhang JB, Cai ZC, Huang XQ (2018). Watermelon planting is capable to restructure the soil microbiome that regulated by reductive soil disinfestation. Appl Soil Ecol.

[39] Liu LL, Huang XQ, Zhao J, Zhang JB, Cai ZC (2019). Characterizing the key agents in a disease-suppressed soil managed by reductive soil disinfestation. Appl Environ Microbiol.

[40] Liu LL, Kong JJ, Cui HL, Zhang JB, Wang FH, Cai ZC, Huang XQ (2016). Relationships of decomposability and C/N ratio in different types of organic matter with suppression of *Fusarium oxysporum* and microbial communities during reductive soil disinfestation. Biol Control.

[41] Liu LL, Xie Y, Zhong X, Deng QQ, Shao Q, Cai ZC, Huang XQ (2023). Facilitating effects of the reductive soil disinfestation process combined with Paenibacillus sp. amendment on soil health and physiological properties of *Momordica charantia*. Front Plant Sci.

[42] Liu LL, Yan Y, Ding HX, Zhao J, Cai ZC, Dai CC, Huang XQ (2021). The fungal community outperforms the bacterial community in predicting plant health status. Appl Microbiol Biotechnol.

[43] Liu SG, García-Palacios P, Tedersoo L, Guirado E, van der Heijden MZA, Wagg C, Chen D, Wang QK, Wang JT, Singh BK, Delgado-Baquerizo M (2022). Phylotype diversity within soil fungal functional groups drives ecosystem stability. Nat Ecol Evol.

[44] Michielse CB, Rep M (2022). Pathogen profile update: *Fusarium oxysporum*. Mol Plant Pathol.

[45] Mishra J, Singh R, Arora NK (2017). Alleviation of heavy metal stress in plants and remediation of soil by rhizosphere microorganisms. Front Microbiol.

[46] Nielsen, M.N., Winding, A., Binnerup, S., Hansen, B., 2022. Microorganisms as indicators of soil health. Neri Technical Report No. 388.

[47] Omirou M, Rousidou C, Bekris F, Papadopoulou KK, Menkissoglou-Spiroudi U, Ehaliotis C, Karpouzas DG (2011). The impact of biofumigation and chemical fumigation methods on the structure and function of the soil microbial community. Microb Ecol.

[48] Paudel BR, Gioia FD, Zhao X, Ozores-Hampton M, Hong JC, Kokalis-Burelle N, Pisani C, Rosskopf EN (2020). Evaluating anaerobic soil disinfestation and other biological soil management strategies for open-field tomato production in Florida. Renew Agr Food Syst.

[49] Prescott CE, Vesterdal L (2021) Decomposition and transformations along the continuum from litter to soil organic matter in forest soils. Forest Ecol Manag 498:119522

[50] Samtani JB, Gilbert C, Weber JB, Subbarao KV, Goodhue RE, Fennimore SA (2012). Effect of steam and solarization treatments on pest control, strawberry yield, and economic returns relative to methyl bromide fumigation. HortScience..

[51] Shao Q, Li XP, Zhao T, Wu YY, Xiang LQ, Pan SF, Guo ZH, Liu LL (2023). Role of reductive soil disinfestation and chemical soil fumigation on the fusarium wilt of Dioscorea batatas Decne suppression. Sustainability -Basel.

[52] Sharma P, Chouhan R, Bakshi P, Gandhi SG, Kaur R, Sharma A, Bhardwaj R (2022). Amelioration of chromium-induced oxidative stress by combined treatment of selected plant-growth-promoting rhizobacteria and earthworms via modulating the expression of genes related to reactive oxygen species metabolism in Brassica juncea. Front Microbiol.

[53] Sun Z, Yang LM, Han M, Han ZM, Yang L, Cheng L, Yang X, Lv ZL (2019). Biological control ginseng grey mold and plant colonization by antagonistic bacteria isolated from rhizospheric soil of *Panax ginseng* Meyer. Biol Control.

[54] Tan XY, Liao HK, Shu LZ, Yao HY (2019). Effect of different substrates on soil microbial community structure and the mechanisms of reductive soil disinfestation. Front Microbiol.

[55] Ullah A, Manghwar H, Shaban M, Khan AH, Akbar A, Ali U, Ali E, Fahad S (2018). Phytohormones enhanced drought tolerance in plants: a coping strategy. Environ Sci Pollut R.

[56] Voltr V, Menšík L, Hlisnikovský L, Hruška M, Pokorný E, Pospíšilová L (2021). The soil organic matter in connection with soil properties and soil inputs. Agron J.

[57] Wang HM, Liu XY, Yang P, Wu RZ, Wang SY, He SN, Zhou QH (2022) Potassium application promote cotton acclimation to soil waterlogging stress by regulating endogenous protective enzymes activities and hormones contents. Plant Physiol Biochem 185:336–343 10.1016/j.plaphy.2022.06.019 35750001

[58] Wen T, Xie PH, Penton CR, Hale L, Thomashow LS, Yang SD, Ding ZX, Su YQ, Yuan J, Shen QR (2022) Specific metabolites drive the deterministic assembly of diseased rhizosphere microbiome through weakening microbial degradation of autotoxin. Microbiome 10:1–15 10.1186/s40168-022-01375-z PMC9587672 36271396

[59] Wang TT, Hao YW, Zhu MZ, Yu ST, Ran W, Xue C, Ling N, Shen QR (2019). Characterizing differences in microbial community composition and function between Fusarium wilt diseased and healthy soils under watermelon cultivation. Plant Soil.

[60] Wei Z, Yang X, Yin S, Shen Q, Ran W, Xu Y (2011). Efficacy of Bacillus-fortified organic fertilizer in controlling bacterial wilt of tomato in the field. Appl Soil Ecol.

[61] Xiong W, Li R, Ren Y, Liu C, Zhao QY, Wu HS, Jousset A, Shen QR (2017). Distinct roles for soil fungal and bacterial communities associated with the suppression of vanilla Fusarium wilt disease. Soil Boil Biochem.

[62] Yan RY, Weiner J, Shi XJ, Wang Y, Zhang RR, Zhu M (2021). Effect of reductive soil disinfestation on the chemical and microbial characteristics of rhizosphere soils associated with *Salvia miltiorrhiza* production in three cropping systems. Appl Soil Ecol.

[63] Yan YY, Wu RN, Li S, Su Z, Shao Q, Cai ZC, Huang XQ, Liu LL (2022). Reductive soil disinfestation enhances microbial network complexity and function in intensively cropped greenhouse soil. Horticulturae..

[64] Yan YY, Xie Y, Zhang JB, Li RM, Ali A, Cai ZC, Huang XQ, Liu LL (2023). Effects of reductive soil disinfestation combined with liquid-readily decomposable compounds and solid plant residues on the bacterial community and functional composition. Microb Ecol.

[65] Zhan Y, Yan N, Miao XY, Li Q, Chen CB (2021). Different responses of soil environmental factors, soil bacterial community, and root performance to reductive soil disinfestation and soil fumigant chloropicrin. Front Microbiol.

[66] Zhang H, Abid S, Ahn JC, Mathiyalagan R, Kim Y, Yang D, Wang YP (2020). Characteristics of *Panax ginseng* cultivars in Korea and China. Molecules.

[67] Zhang T, Gao Y, Han M, Yang LM (2021). Changes in the physiological characteristics of *Panax ginseng* embryogenic calli and molecular mechanism of ginsenoside biosynthesis under cold stress. Planta..

[68] Zhao J, Li YL, Wang BY, Huang XQ, Yang L, Lan T, Zhang JB, Cai ZC (2017). Comparative soil microbial communities and activities in adjacent Sanqi ginseng monoculture and maize-Sanqi ginseng systems. Appl Soil Ecol.

[69] Zhao J, Liu SZ, Zhou X, Xia Q, Liu X, Zhang SR, Zhang JB, Cai ZC, Huang XQ (2020). Reductive soil disinfestation incorporated with organic residue combination significantly improves soil microbial activity and functional diversity than sole residue incorporation. Appl Microbiol Biotechnol.

[70] Zhao J, Zhou X, Jiang AQ, Fan JZ, Lan T, Zhang JB, Cai ZC (2018). Distinct impacts of reductive soil disinfestation and chemical soil disinfestation on soil fungal communities and memberships. Appl Microbiol Biotechnol.

[71] Zhou YJ, Li JH, Friedman CR, Wang HF (2017). Variation of soil bacterial communities in a chronosequence of rubber tree (Hevea brasiliensis) plantations. Front Plant Sci.

