# Supplementary material for: Facilitating Effects of Reductive Soil Disinfestation on Soil Health and Physiological Properties of Panax ginseng
Source: Microb Ecol. 2024 Mar 21;87(1):54. doi: 10.1007/s00248-024-02349-4 (PMC10957680; doi:10.1007/s00248-024-02349-4)
Supplement: Supplementary file 1 [file 248_2024_2349_MOESM1_ESM.docx]

**Supporting Information**

**Facilitating effects of the reductive soil disinfestation and chemical soil fumigation on soil health and physiological properties of *Panax ginseng***

Yu Zhan^a^, Ergang Wang^a^, Yi Zhou^b^, Guixiang He^a^, Pengyuan Lv^a^, Lixiang Wang^b^, Tingting Zhou^a^, Xinyue Miao^a^, Changbao Chen^a,^*, Qiong Li^a,^*

^a^ Jilin Ginseng Academy, Changchun University of Chinese Medicine, Changchun, 130117, China

^b^ School of Pharmaceutical Sciences, Changchun University of Chinese Medicine, Changchun, 130117, China

***Corresponding author**

Qiong Li Tel: +86 17743140380; E-mail: wode17k@163.com

Changbao Chen Tel: +86 13504418096; E-mail: [ccb2021@126.com](mailto:ccb2021@126.com)

**Table S1**. Number of sequences, OTUs, and coverage (only after re-sampling) after quality control and re-sampling of soil samples from different treatments.

| Treatment |  | After quality control | |  | After re-sampling | | |
| --- | --- | --- | --- | --- | --- | --- | --- |
|  |  | Sequences | OTUs |  | Sequences | OTUs | Coverage |
| CK | Rep1 | 71056 | 480 |  | 67955 | 413 | 0.997 |
|  | Rep2 | 73521 | 451 |  | 67955 | 386 | 0.998 |
|  | Rep3 | 69702 | 484 |  | 67955 | 423 | 0.997 |
|  | Rep4 | 68899 | 443 |  | 67955 | 380 | 0.997 |
| CSF | Rep1 | 69183 | 195 |  | 67955 | 160 | 0.999 |
|  | Rep2 | 69729 | 195 |  | 67955 | 158 | 0.999 |
|  | Rep3 | 67955 | 154 |  | 67955 | 139 | 0.999 |
|  | Rep4 | 69372 | 158 |  | 67955 | 135 | 0.999 |
| RSD | Rep1 | 69102 | 530 |  | 67955 | 473 | 0.997 |
|  | Rep2 | 71181 | 442 |  | 67955 | 430 | 0.997 |
|  | Rep3 | 71315 | 518 |  | 67955 | 456 | 0.997 |
|  | Rep4 | 72691 | 537 |  | 67955 | 487 | 0.997 |

**Table S2**. Weighted_unifrac ADONIS difference analysis of soil fungal beta diversity between different treatments.

| Diffs | Df | SumsOfSqs | MeansSqs | Fvalue | R^2^ | Pvalue | Significant |
| --- | --- | --- | --- | --- | --- | --- | --- |
| CK-vs-CSF | 1 | 1.6748 | 1.6748 | 8.6542 | 0.5906 | 0.027 |  |
| CK-vs-RSD | 1 | 0.4588 | 0.4588 | 4.4686 | 0.4269 | 0.027 |  |
| CSF-vs-RSD | 1 | 1.4084 | 1.4084 | 9.5098 | 0.6131 | 0.027 |  |
| CK-vs-CSF-vs-RSD | 2 | 2.3613 | 1.1807 | 7.9722 | 0.6392 | 0.001 | *** |

**Table S3**. Weighted_unifrac ANOSIM difference analysis of soil fungal beta diversity between different treatments.

| Diffs | Rvalue | Pvalue | Significant |
| --- | --- | --- | --- |
| CK-vs-CSF | 0.9896 | 0.034 |  |
| CK-vs-RSD | 0.6354 | 0.034 |  |
| CSF-vs-RSD | 0.7396 | 0.034 |  |
| CK-vs-CSF-vs-RSD | 0.7477 | 0.001 | *** |

**Table S4**. Top ten keystone taxa of microbial community networks between different treatments.

| CK | CSF | RSD |
| --- | --- | --- |
| *Mortierella*  (OTU384) | *Scytalidium*  (OTU580) | *unclassified_f__Lasiosphaeriaceae*  (OTU376) |
| *Trichocladiu*  (OTU1092) | *unclassified_p__Ascomycota*  (OTU675) | *Gibberella*  (OTU241) |
| *Mortierella*  (OTU759) | *Byssochlamys*  (OTU629) | *Mortierella*  (OTU384) |
| *Mortierella*  (OTU1127) | *Cutaneotrichosporon*  (OTU604) | *Tausonia*  (OTU12) |
| *Lectera*  (OTU241) | *Issatchenkia*  (OTU645) | *Cladorrhinum*  (OTU436) |
| *Lectera*  (OTU899) | *Thermomyces*  (OTU583) | *Tausonia*  (OTU874) |
| *Tausonia*  (OTU905) | *unclassified_k__Fungi*  (OTU682) | *Neocosmospora*  (OTU23) |
| *Tausonia*  (OTU12) | *Thermoascus*  (OTU642) | *unclassified_o__Glomerellales*  (OTU745) |
| *Neonectria*  (OTU752) | *Monascus*  (OTU661) | *Solicoccozym*a  (OTU407) |
| *Gibberella*  (OTU439) | *Neocosmospora*  (OTU23) | *Gibberella*  (OTU439) |

**
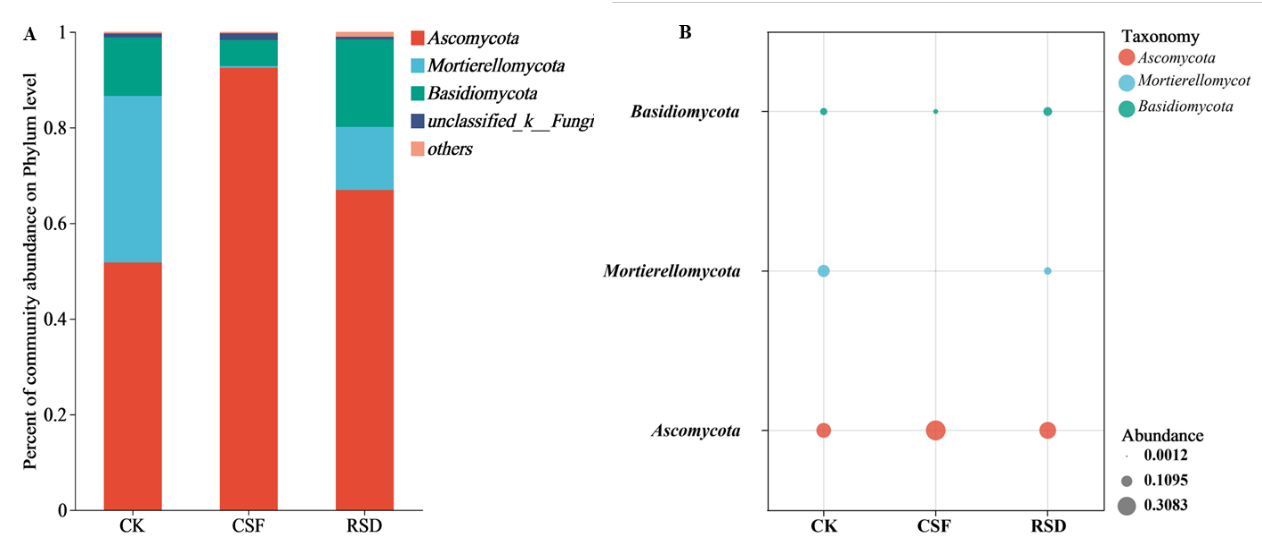
**

**Fig. S1** Taxonomic composition of fungal communities in soils under different treatments at the phylum level (A) and abundance analysis (*P* < 0.05) of the three most abundant phyla in soils under different treatments (B).


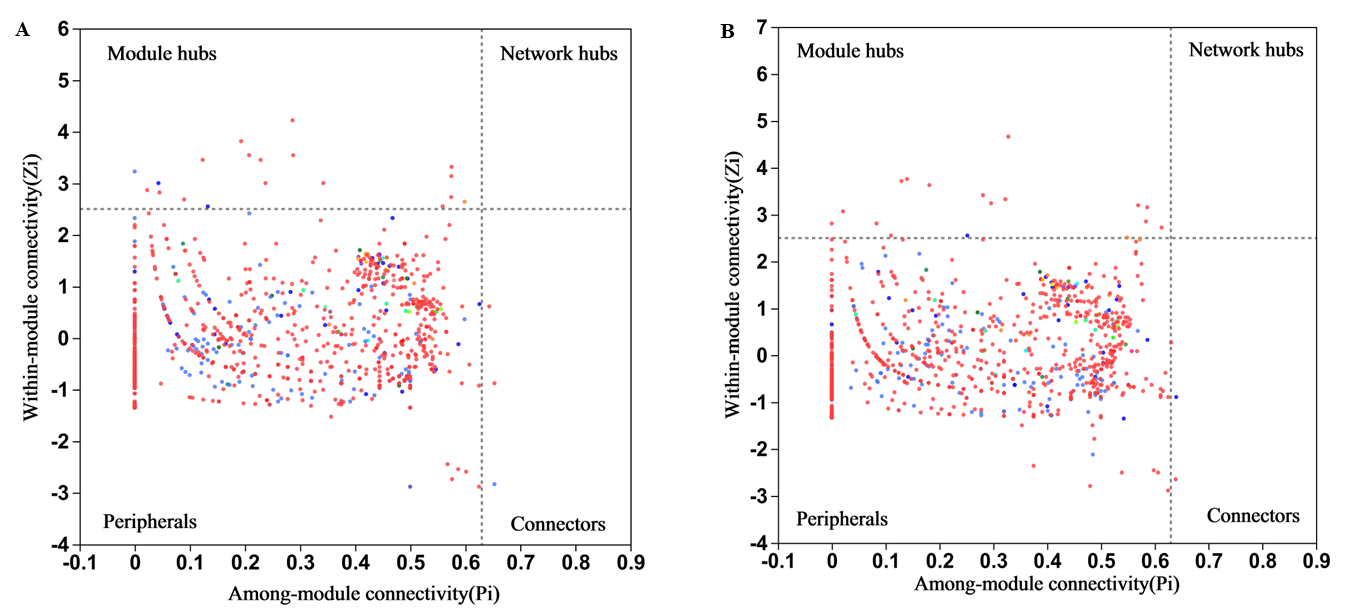


**Fig. S2** Zi-Pi plot shows the distribution of OTUs of fungal communities and functions based on their topological roles with different treatments.
